# An underexploited invisible gold resource in the Archean sulphides of the Witwatersrand tailings dumps

**DOI:** 10.1038/s41598-023-30219-5

**Published:** 2023-02-22

**Authors:** Steve Jason Chingwaru, Bjorn Von der Heyden, Margreth Tadie

**Affiliations:** 1 Department of Earth Sciences, Stellenbosch University, Private Bag X1, Matieland, 7602 South Africa; 2 Department of Process Engineering, Stellenbosch University, Private Bag X1, Matieland, 7602 Stellenbosch South Africa

**Keywords:** Geochemistry, Geology, Mineralogy, Sedimentology, Environmental sciences, Solid Earth sciences, Engineering, Chemical engineering

## Abstract

The tailings dumps originating from gold mining in South Africa’s Witwatersrand still contain notable gold endowments. Most tailings reprocessing operations target a native gold fraction using re-milling and carbon-in-leach extraction; however, up to 50–70% of the remaining gold is still not recoverable and instead discarded to the re-dump stream along with abundant sulphides. The mineralogical deportment of this unrecoverable gold underwent a detailed investigation. Using in situ laser ablation ICP-MS mineral chemistry measurements, we show that this gold that is inaccessible to conventional recovery is hosted preferentially in pyrite and arsenian pyrite. Importantly, complementary optical and electron microscopy observations reveal that the rounded detrital forms of these minerals contain the highest gold concentrations (0.01–2730 ppm), showing some similarity to values reported for sulphides from primary orogenic gold deposits found in surrounding Archean-aged granite-greenstone belt remnants. We suggest that detrital auriferous sulphides have been overlooked by historical primary and secondary beneficiation, and thus represent a large (up to 420 tons Au) and under-exploited Au resource currently residing in easily-mined (surficial) Witwatersrand tailings dumps. We further suggest that targeted re-mining of sulphide mineral fraction has the potential to improve gold recovery, recuperate ‘sweetener’ by-product metals (e.g. Cu, Co, Ni) and directly eliminate heavy metal pollution and acid mine drainage issues associated with surficial tailings dumps.

## Introduction

**Figure 1:**
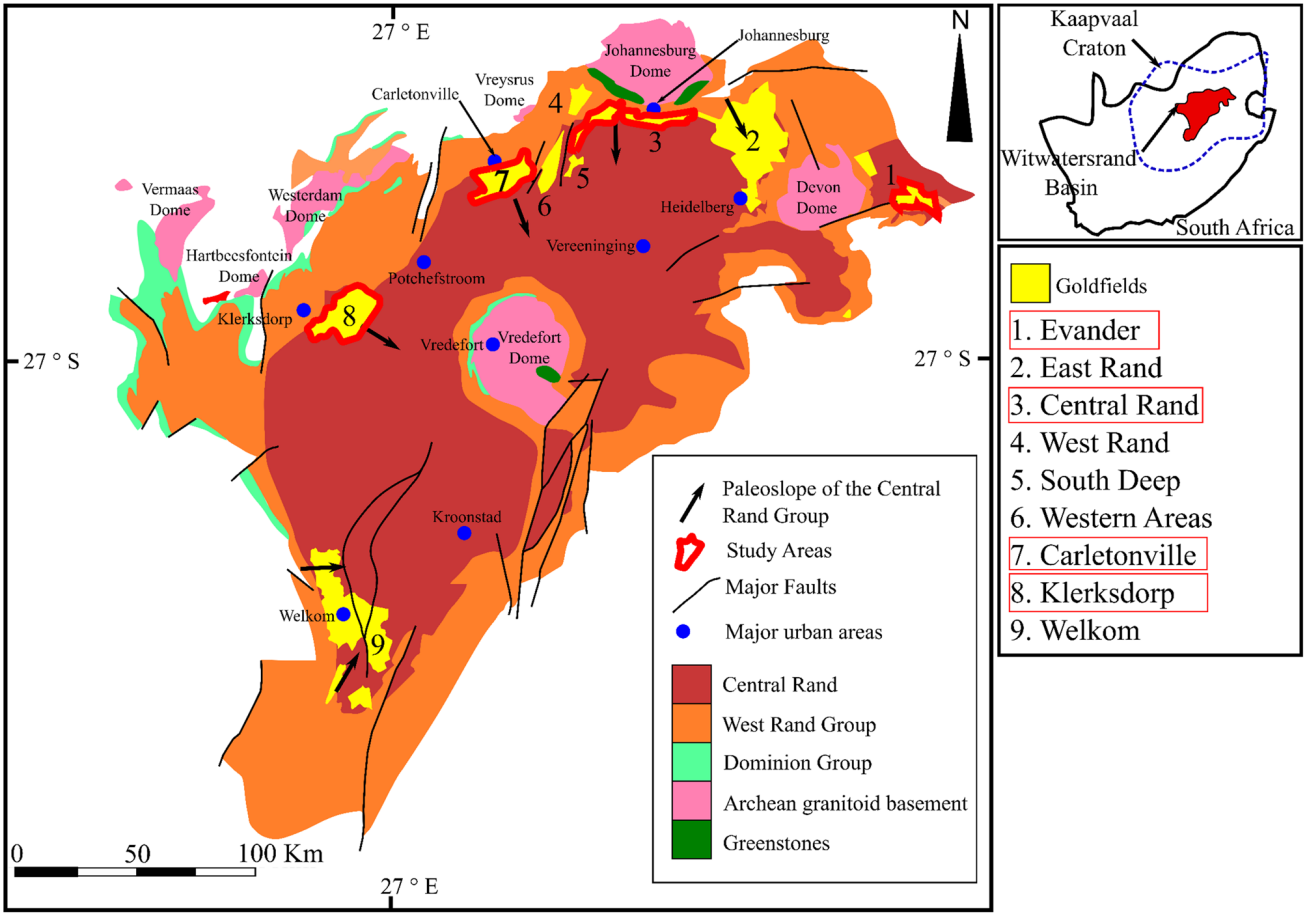
A surface geological map of the Witwatersrand basin, displaying the goldfields referred to in the text. The inset diagram shows the position of the Witwatersrand basin relative to the margins of the Archean Kaapvaal craton within South Africa modified after Frimmel et al*.*^59^. Since the figure is modified after a Society of Economic Geologists publication, it is under fair use therefore no formal permission is required for this map. Please see link: https://www.segweb.org/SEG/Publications/Copyright-and-Open-Access/SEG/Publications/Copyright-and-Open-Access.aspx?hkey=c614a0ac-cff3-4d7d-8cb3-39008ea46c24. The figure was modified by Inkscape an open source scalable vector graphics editor, version 1.0.2–2 with URL: https://inkscape.org/.

The Witwatersrand is a 2.9–2.8 Ga sedimentary basin located on the Kaapvaal Craton (Fig. 1)^1^. The Witwatersrand basin together with the gold reefs from the Venterdorp and Transvaal supergroups is world-renowned for its super-giant gold endowment. The gold in the basin is largely associated with a series of quartz-pebble conglomerate beds (reefs) that formed during continental sedimentation in alluvial fans and braid-plain depositional environments^2^. Historical gold production from the Witwatersrand sedimentary basin accounts for ~ 28% of the total gold that has ever been mined globally^3^. Despite the quantitative importance of the Witwatersrand deposits, the ultimate provenance of the gold is still a topic of scientific debate, in part because any obvious hinterland gold source has been removed from the geological record through erosional processes^4,5^. Genetic models for the formation of the mineralisation include solution transport models with algal mat ‘traps’^3,6,7^, placer models^8^, hydrothermal models^9,10^, and the ‘modified-placer’ model^11–13^. Perhaps the most broadly-accepted theory is this modified-placer model since it combines the microscale textural observations of the hydrothermal model with the observation that mined gold grades follow spatial distributions that are better explained by the placer model. Briefly, this model suggests that native gold grains, likely sourced from continental-scale chemical and mechanical weathering and erosion of granite-greenstone-dominated hinterlands^3,14^, were initially concentrated in conglomerates along with other dense detrital minerals. Subsequent hydrothermal fluid overprints resulted in local-scale remobilisation of gold within these conglomeratic units, thus accounting for the observed crystalline gold textures e.g.^15^ and sulphide morphologies e.g.^16^.

Sedimentation of the Witwatersrand Supergroup occurred under a reducing Archean atmosphere and modern-style plate tectonics, as evidenced by the presence of detrital pyrite (FeS_2_) detrital uraninite (UO_2_) and macro-diamonds within the auriferous quartz pebble conglomerates^17,18^. Given the possibility that Witwatersrand gold was derived from orogenic-type primary gold deposits in an Archean granite-greenstone hinterland, it is reasonable to infer that a proportion of the detrital pyrite may similarly have been sourced from such deposits. Remnant vestiges of Archean greenstone belts located on the Kaapvaal craton commonly show elevated gold endowments (e.g. Barberton Greenstone belt = 376 t gold; Amalia-Kraaipan Greenstone belt = 32 t gold; Giyani Greenstone belt = 12 t gold; Pietersburg Greenstone belt = 4 t gold^19^). Much of this gold can be classified as ‘*invisible gold*’, a loosely-defined term reserved for gold that behaves in a refractory manner to direct cyanidation^20^, which encompasses both gold dissolved in the sulphide mineral structures (generally arsenopyrite and arsenian pyrite) as a cation substituent^21,22^, and gold occurring as submicron and nano-scale inclusions within sulphide mineral hosts^23^. For example, arsenopyrite and arsenian pyrite mined as part of the run-of-mine from orogenic gold shoots in the Barberton greenstone belt commonly host high concentrations of invisible gold (up to 1000 ppm^24^), and the prevalence of this ore type has warranted the use of biological oxidation (BIOX®) beneficiation strategies^25^. Similarly, fine and invisible gold mineralization is reported in the Kraaipan Greenstone Belt, strongly associated with pyrite and pyrrhotite^26,27^.

The mining of Witwatersrand conglomerates and their contained gold and associated detrital sulphides dates back to 1885^28^. This extended mining legacy has resulted in a massive accumulation of tailings material (6 billion tons^29^) which, largely on account of historical processing inefficiencies, is currently being re-mined as a secondary gold resource^30,31^. Most of the dedicated plants that reprocess Witwatersrand tailings material apply re-milling followed by carbon-in-leach extraction as the beneficiation strategy. This strategy targets native gold and results in a gold recovery that ranges between 30 and 50%^30–32^. The present contribution seeks to better understand the mineralogical deportment of the remaining 50–70% of the gold that is deemed refractory to this beneficiation strategy, with particular emphasis on the role of Archean detrital pyrite as a viable mineral host for the ‘missing’ recovery.

### Gold deportment in density-defined mineral fractions and minerals

**Figure 2:**
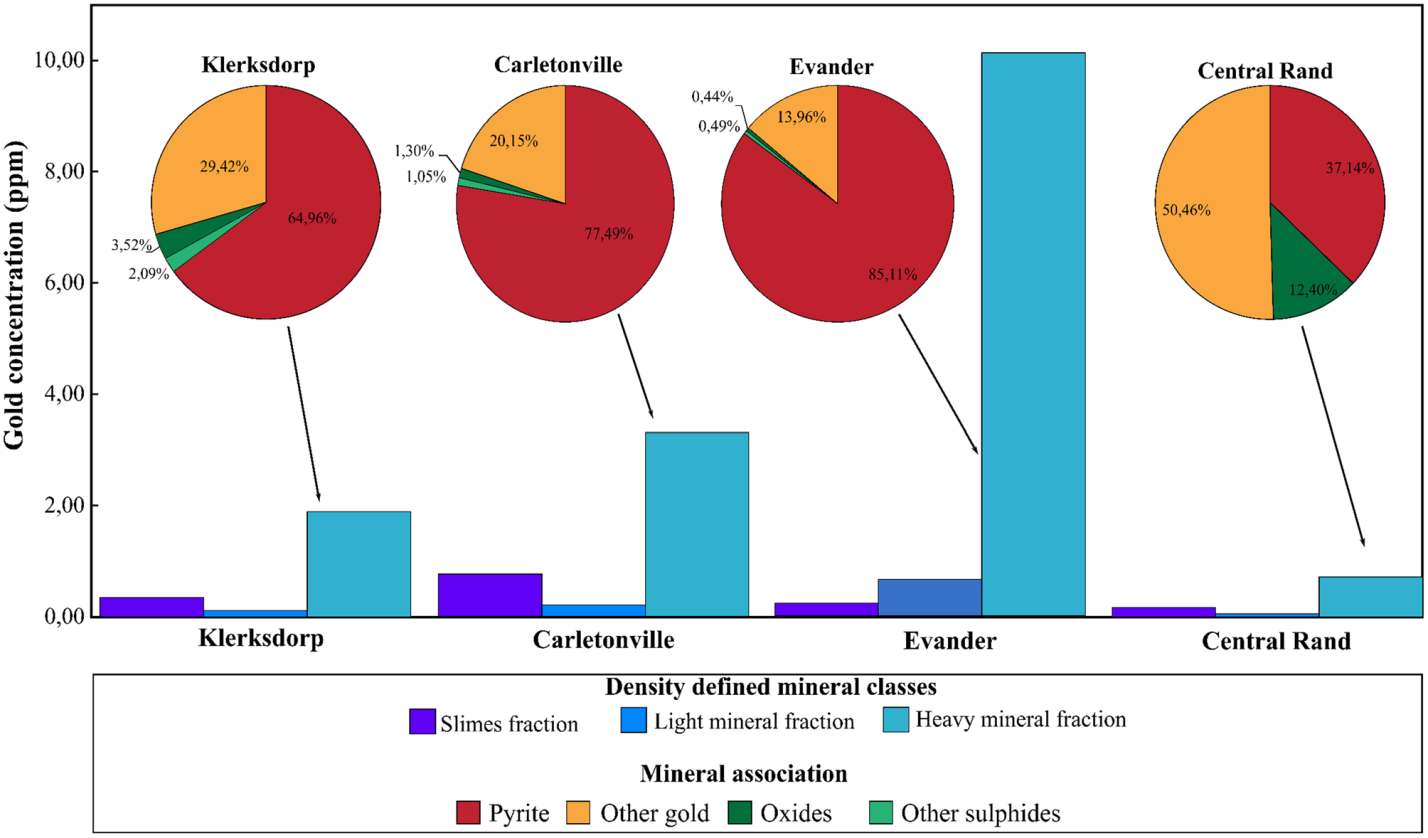
A bar graph showing the gold concentration and mass percentages within each of the density-defined operational mineral fractions from the corresponding goldfield tailings dump material. The pie charts display the distribution of gold by mineralogy within the heavy mineral fraction of the tailings material. Other sulphides refer to arsenopyrite, galena, sphalerite, chalcopyrite, pentlandite and pyrrhotite. Oxides refer to hematite, magnetite, and limonite/goethite. The other gold may represent sub-microscopic free grains, gold that is associated with minerals that were not analysed using LA ICP-MS or a manifestation of the error propagation through the mass balance calculations.

Tailings material from four regionally-distributed Witwatersrand tailings dumps (Carletonville, Central Rand, Evander and Klerksdorp goldfields; Fig. 1) was pre-concentrated into density-defined mineral fractions, providing the first step toward developing a statistical understanding of the main gold carriers. Results from aqua regia digestion and gold assay consistently show that the heavy mineral fraction (ρ > 2,95 g.cm^−3^; size 15–75 µm) hosts the highest measured gold concentrations which range between 0.71 and 10.12 ppm (Fig. 2). This accounts for 0.89–13.23% for the mass of gold in the samples. The light mineral fraction (d < 2.95 g.cm^−3^; size 15–75 µm) and slimes fraction (< 10 μm) have measured gold concentrations that are an order of magnitude lower, measuring across the four goldfields between 0.05–0.22 ppm and 0.17–0.76 ppm respectively. This preferential partitioning of gold into the heavy mineral fraction may be explained by the presence of nano-particulate native gold grains, or by a chemical association between gold and heavy gangue minerals such as the sulphides and oxides^20,33,34^. Our detailed microscopic investigation of the heavy mineral fraction using optical microscopy (40X magnification; resolution = 20 µm), and electron microscopy (1345X magnification; resolution = 40–10 µm) did not reveal any free gold particles. Instead, the heavy mineral fraction comprises of gangue mineralogy: 13–44% silicates; 35–76% sulphides; 9–20% oxides and 0.01–0.34% carbonates and phosphates. This suggests that the assayed gold must reside as ‘invisible’ gold within the sulphide and oxide minerals.

Extensive in-situ laser ablation analyses (800 spots, spot size = 30 µm) and gold mineralogical balancing which refers to determining the mineralogical deportment of gold, reveal that arsenian pyrite and pyrite account for most of the gold within the heavy mineral fraction. Specifically, these two minerals account for approximately 65% of gold in the Klerksdorp heavy mineral fraction, 78% of gold in the Carletonville heavy mineral fraction and 85% of gold in the Evander heavy mineral fraction (Fig. 2). Measurements of the Central Rand heavy mineral fraction indicate that it is an outlier showing low gold deportment to the sulphides (37%), which may be an artefact of the low total gold concentration and a limited number of sulphide grains (two factors that may be codependent in tailings material). Gold deportment into goethite/limonite, hematite and other sulphide minerals is quantitatively less important (i.e. typically accounting for ~ 1–6% of total gold in the heavy mineral fraction), with the largest concentrations observed in gothite/limonite (0.78 ppm) hematite (0.64 ppm), and pyrrhotite (0.34 ppm) respectively.

### Petrographic and chemical characterization of auriferous pyrite

**Figure 3:**
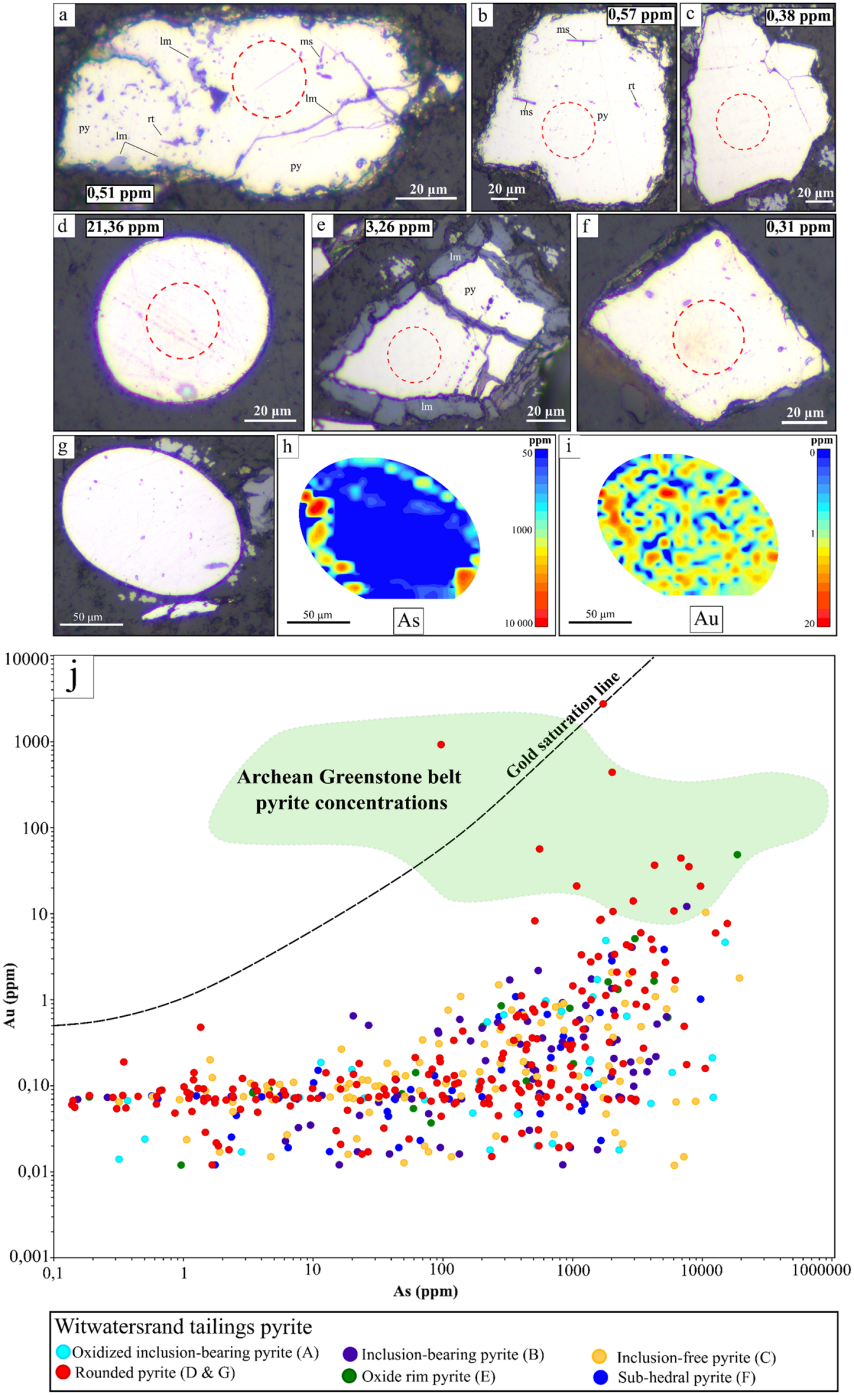
(**a-h)** Optical images of the six pyrite types classified according to morphology and texture that are found within the heavy mineral fraction of the Witwatersrand tailings dumps. The ablation spot on the grain and the resultant mean gold concentrations are also presented. (**a**) An oxidized inclusion-bearing anhedral pyrite with inclusions of muscovite, rutile and limonite. (**b**) A fresh inclusion-bearing anhedral pyrite (**c**) A fresh inclusion-free anhedral pyrite (**d**) A fresh rounded anhedral pyrite (**e**) An oxide rim anhedral pyrite. (**f**) A fresh cubic inclusion-free euhedral/subhedral pyrite. (**g**) Shows a rounded detrital pyrite grain from the tailings dump material with corresponding LA ICP-MS maps of (**h**) gold and (**i**) arsenic. (**j**) A logarithmic plot of the concentration relationship between gold and arsenic within the different classes of pyrite from the heavy mineral fraction of the Witwatersrand tailings dump material. The gold/arsenic concentration field of auriferous pyrite from gold reefs in the Barberton Greenstone Belt has also been plotted^24,57,58^. The gold saturation line proposed for pyrite in gold deposits represents the maximum limitations for gold in solid solution within the pyrite structure, pyrite plotting above the line will have gold as free gold inclusions^22^.

The observation that pyrite is the preferred mineral host for unrecovered gold in Witwatersrand tailings material demands a further level of classification to discern between auriferous and non-auriferous pyrite. Based on morphology, texture, and degree of oxidation (from surface weathering) six classes of pyrite were designated: viz. 1. inclusion-bearing anhedral 2. inclusion-free anhedral, 3. oxidized inclusion-bearing anhedral 4. oxide rim anhedral 5. rounded anhedral and 6. Fresh inclusion-free euhedral/subhedral (Fig. 3a–f). Given that the tailings materials are ex-situ samples that have been subject to extensive comminution, processing and surface weathering, this coarse classification allows only limited comment on the ultimate provenance of the pyrite (e.g. detrital). Notwithstanding this limitation, we believe that the distinctive rounding of pyrite particles from the ‘rounded pyrite’ class (Fig. 3d,h–j) is a natural feature developed during fluvial processing and sedimentation of the Witwatersrand conglomerates. In previous in-situ morphological studies of Witwatersrand conglomerate-hosted pyrite, these distinctively rounded textures have been used to diagnostically characterize pyrite as detrital in origin e.g.^16,35^.

Figure 3j presents a binary plot of arsenic concentration versus gold concentration measured for individual LA ICP-MS ablation spots located within pyrite grains from all six different pyrite classes (Fig. 3a–g). The pyrite morphological class that hosts the highest gold concentrations is the rounded, nominally-detrital pyrite class, which records an average spot concentration value of 21.36 ppm gold and a maximum of 2700 ppm gold. This maximum data point and the second most auriferous pyrite data point both plot above the gold saturation line of Reich et al*.*^22^, indicative that the gold is occurring within these pyrite as nanoparticles (or sub-micron particles) of free gold (Supplementary Information 1a). Importantly, a further eleven detrital pyrite plots below the gold saturation line but above the concentration value of 10 ppm gold, representing 5.74% of all the detrital pyrite analyzed, and 2.43% of the total dataset. Laser ablation mapping of five individual detrital pyrite grains shows that the gold may be associated with pyrite rims (e.g. Fig. 3 h–j) or more variably distributed throughout the grains, and that there is commonly a strong association with arsenic (Fig. 3h–j^22,36,37^). In contrast to the detrital pyrite population, only one pyrite particle from the oxide rim pyrite class and only one particle from the inclusion-bearing pyrite class fall above the arbitrarily-assigned 10 ppm concentration cut-off. Previous research has been conducted on the pyrite from the Witwatersrand Basin’s Carbon Leader and Upper Elsbury Reefs using in-situ ablation e.g.^12,27,38^. Our data, collected from the representative tailings samples, has a general overlap with pyrite data from these previous works however, higher gold concentrations have been reported by Large et al*.*^12^ and Large & Maslennikov^38^ largely associated with diagenetic pyrite overgrowths on detrital pyrite rims (see the comparison in Supplementary Information 2). Moreover, considering published data for pyrite sampled from orogenic gold shoots in the Barberton greenstone belt (assumed to be representative of a possible granite-greenstone hinterland gold source),  ~ 5% of the detrital pyrite shows an overlap in terms of their gold/arsenic concentration signatures (Fig. 3j;^24^). The possible linkages between Witwatersrand pyrite and an Archean greenstone orogenic gold source region were further tested using multivariate analyses of pyrite mineral chemistry, revealing some overlap relationships within a principal component biplot (Supplementary Information S3).

### Auriferous detrital pyrite in tailings as a surface gold resource

The advent of Witwatersrand gold mining (137 years ago) greatly predates the development of modern analytical techniques capable of quantifying mineral chemistry at ppm concentration levels (e.g. LA ICP-MS). This temporal disconnect may have contributed to the entrenched paradigm that the Witwatersrand is purely a native gold resource despite (1) The early^14,39^ and still prevalent^16–41^ hypothesis that greenstone belt orogenic gold mineralization may represent the ultimate provenance of Witwatersrand gold; (2) a firmly established paragenetic relationship between gold and pyrite/arsenopyrite in orogenic gold mineralization studied in greenstone belt vestiges on the Kaapvaal craton^35,42^; and (3) knowledge that these sulphides behaved as stable heavy minerals during sedimentation under the reducing Archean paleo-environmental conditions e.g.^17,39^. Here we highlight the presence of auriferous detrital pyrite identified within the Witwatersrand tailings material, which commonly shows geochemical signatures that are comparable to those reported for pyrite associated with orogenic gold in Kaapvaal greenstone belts. Implications of this are two-fold: firstly, exploration and mining have specifically targeted free gold (19.3 g.cm^−3^) in conglomeratic units, whereas our results suggest that there may be exploration potential for an accumulation of detrital auriferous pyrite (~ 5 g.cm^−3^) in alternative (discrete) sedimentary settings. The gold grades of such a sedimentary setting may however be restrictively low to warrant mining. Secondly, historically mined detrital auriferous pyrite has been overlooked during original processing as well as during more recent reprocessing. This ‘invisible’ gold is known to be inaccessible through direct cyanidation^20^, and may thus be a significant contributor to the 50–70% recovery inefficiencies associated with secondary beneficiation of the Witwatersrand tailings. From an economic standpoint, extraction of auriferous pyrite from these tailings is deemed more favourable due to fine grain sizes and surficial location, respectively negating comminution and the high overheads associated with underground mining.

### Environmental benefits of targeted re-mining of detrital pyrite

A metallurgical flowsheet that targets the sulphide fraction during tailings reprocessing that includes either a microbial-assisted heap leaching process or sulphide concentration phase by froth flotation subsequent pre-oxidative treatment or followed by gold leaching (see recommendations by^43–45^) would recover this invisible gold fraction along with other metals and metalloids that may be of economic interest or environmental concern. Such a beneficiation strategy would be highly beneficial to the Witwatersrand region, where acid mine drainage (AMD), and concomitant release and mobility of deleterious elements (e.g. Cu, Cd, Co, Pb, Zn, As, Ni, Hg, U and SO_4_^2−^), is an environmental issue^46–49^. This is because sulphide oxidation under surface weathering conditions (e.g.Fig. 3b,f) is the primary cause of acid mine drainage pollution in the Witwatersrand region^48^.

**Figure 4:**
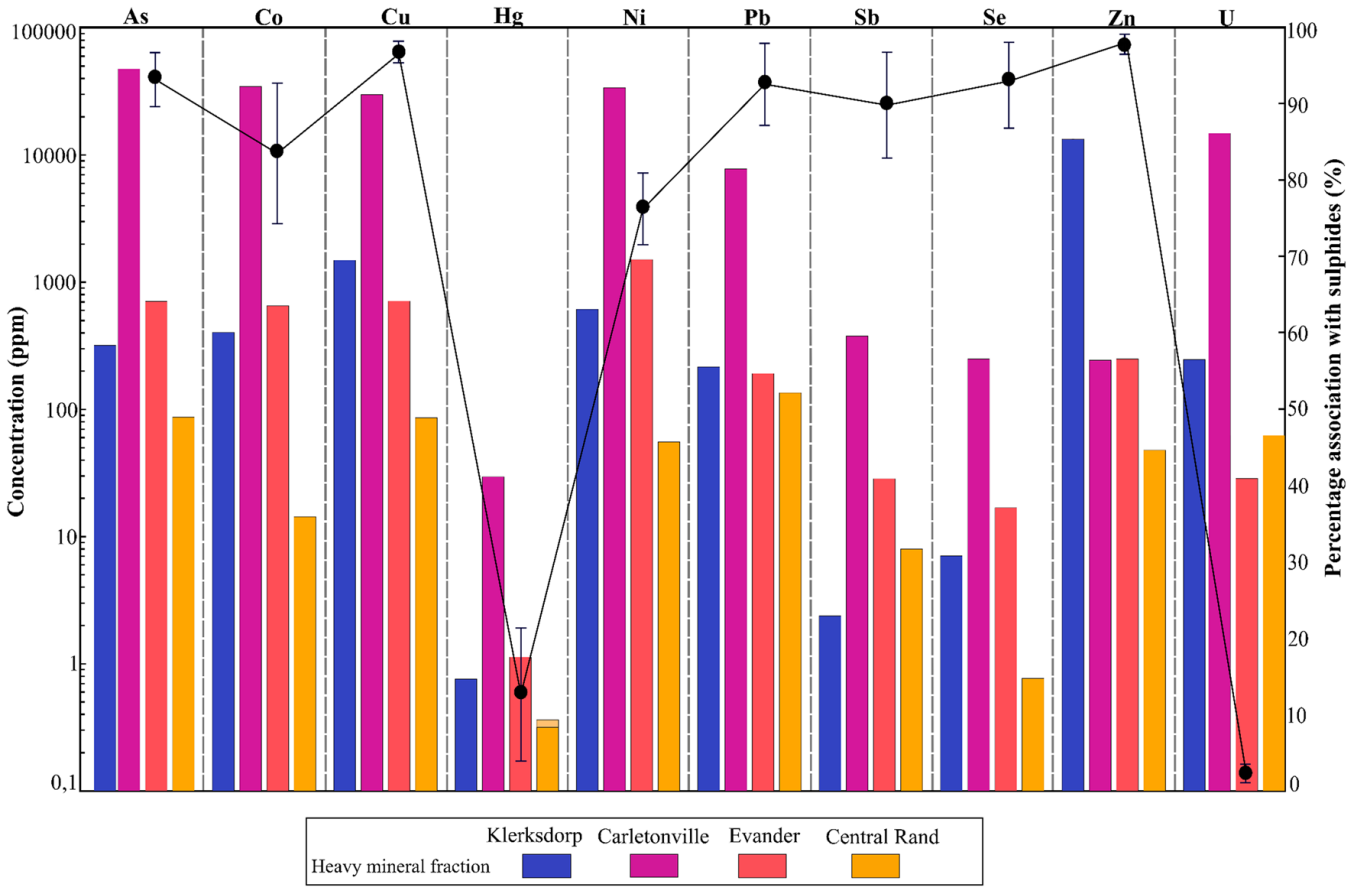
A bar chart with the concentrations of deleterious elements hosted within the heavy mineral fraction from the different goldfield tailings dumps of the Witwatersrand Basin. A line graph is plotted with the concentration bar chart to represent the average percentage of deleterious elements deporting to the sulphide fraction within the heavy mineral fraction from the different tailings dumps study areas. Error bars represent one standard deviation resulting from averaging across all four gold fields (each goldfield comprising of four samples).

Most deleterious elements in Witwatersrand tailings are found preferentially in the heavy mineral fraction (Fig. 4), whereas the light mineral fractions have deleterious metal concentrations that are relatively low with the majority complying with established re-dump criteria^50,51^. Results from the in situ LA ICP-MS analyses and mass balance calculations reveal that within the heavy mineral fraction, most of the deleterious elements deport to the sulphide mineral fraction (Fig. 4). For example, the sulphides host approximately 95% of Zn, Cu, As and Pb; 55–97% of Ni; 64–99% of Co; 77–99% of Se and 84–99% of Sb within the heavy mineral fraction. This observed deportment of the high deleterious element concentrations specifically within the tailings sulphides strongly underpins an environmental motivation towards sulphide removal during tailings reprocessing. The value proposition of such an approach would be further enhanced by the recovery of high-demand heavy metals such as Co, Cu and Ni as by-products, which respectively have average calculated concentrations of 9755.88 ± 2330 ppm Co, 8771.40 ± 1817 ppm Cu and 9822.57 ± 1810 ppm Ni within the sulphide mineral fraction.

## Conclusion

The study highly suggests that the fraction of gold inaccessible to conventional processing technologies in the Witwatersrand tailings ore material is attributed to a percentage of invisible gold, predominately within the arsenian pyrite and pyrite. Preserved rounded detrital pyrite classes within the tailings dump material exhibit the highest concentration of gold, with a maximum gold concentration similar to auriferous pyrite from existing granite-greenstone belt gold deposits. The residual sulphides in the tailings dump potentially represent a large underexploited economic resource of up to 420 tons of gold, in which pre-treatment beneficiation can directly improve gold recovery by ~ 22% during secondary reprocessing. This will further alleviate the growing ramifications of AMD and effluents with deleterious elements in the Witwatersrand mining region, while potentially recovering additional high-demand by-product metals.

## Methods

### Bulk sampling and sample preparation

Duplicates of 150 kg of sample material were acquired from four tailings dams storage facilities associated with mining in the Evander, Carletonville, Central Rand and Klerksdorp Goldfields of the Witwatersrand Basin (Fig. 1). To ensure sample representativity and homogeneity, sub-sampling comprised blending via a cone and quarter, rifle and rotary splitting protocols.

### Pre-concentration and mount preparation

Approximately 1 kg from each of the bulk samples underwent a series of modified density separation techniques as prescribed by Zhou & Cabri^52^, to create density-defined mineral fractions. The applied techniques were decantation (desliming), superpanning and a two-stage heavy liquid separation using tetrabromoethane at 2.95 g/cm^3^ density and lithium metatungstate at 2.71 g/cm^3^ density. The resultant density-defined mineral fractions were a slimes fraction (particle size < 25 μm), a light mineral fraction and a heavy mineral fraction. This sample preparation approach ensures a statistically representative and unbiased acquisition of data by subsequent analysis techniques. Approximately 0.3 g of representative aliquots from each of the density-defined mineral fractions was placed as a monolayer in an epoxy mount, and subsequently diamond polished to a 1 μm finish.

### Sample analyses

#### Aqua regia digestion & ICP-MS analysis

Aqua regia digestion and inductively coupled plasma mass spectrometry (ICP-MS) analyses were conducted to determine the bulk trace element concentrations (As, Au, Co, Cu, Hg, Ni, Pb, Sb, Se, Zn & U). This was done on approximately 0.55 g duplicates from each bulk sample, each density fraction and on three blank samples at the Central Analytical Facilities (CAF), Stellenbosch University. Aqua regia digestion comprised of ± 1 g of sample material partially digested in a (3:1) acid mixture of 6 ml nitric acid and 2 ml of supra pure hydrochloric acid. The sample material is digested with certified reference material WQB-1 and PTM-1a The sample solution was then heated in a MARS microwave digester at 1600W power level for 4–6 h at temperatures of approximately 60 °C to ensure the near-total breakdown of aqua regia soluble minerals (see aqua regia digestible minerals^53,54^). After the containers cooled, they underwent dilution to a volume of 50 ml before quantitative analysis. A total of 20 elements were analysed using an Agilent 7900 ICP -MS at 1600 W power level, 0.83 L/min argon as the carrier gas, 0,15 L/min make-up gas flow rate, 5–6 ml/min of helium and hydrogen flow rate and a multi-elemental calibration standard for confidence in ICP-MS readings.

### Image analysis via QEMSCAN, SEM & Optical microscopy

Optical microscopy was used for scanning visible gold grains, describing the minerals reporting to each of the density-defined operational mineral fractions and classifying the different pyrite classes hosted in the heavy mineral fraction. Preliminary electron microscopy was conducted at Stellenbosch University’s Central Analytical Facilities (CAF) using a Zeiss Merlin FE-SEM was utilized at conditions of 10 kV voltage, stage distance of 9.55 mm, probe current of 11 nA and equipped with Oxford INCA software.

Each heavy mineral fraction mount underwent analysis with the QEMSCAN at the University of Cape Town. This analytical technique was incorporated into the study to determine bulk mineral percentages of the heavy mineral fraction, which in turn was used to validate the assay data derived from acid digestion and for mass balance calculations. Additionally, a false image colour map was generated to assist with the identification of ‘visible gold’ and potential gold carriers, as well as a visual aid to identify potential grains for subsequent LA ICP-MS analysis. The QEMSCAN operation involved Bruker energy dispersive spectroscopy (EDS) detectors at 20 kV accelerating voltage and a 10 nA beam current. Particle mineral analyses were done at 3–5 μm and 211 × magnification.

### In-situ laser ablation inductively coupled mass spectrometry (LA ICP-MS)

LA ICP-MS was carried out on 800 grains of oxides (hematite, goethite/limonite, rutile) and sulphides (pyrite, arsenopyrite, galena, sphalerite, chalcopyrite, pentlandite and pyrrhotite) from the heavy minerals fraction mounts using a grid method at CAF, Stellenbosch University with a Resolution 193 nm Excimer laser from Applied spectra, coupled with an Agilent 7700 Q ICP MS. This was done to obtain concentrations of trace elements (Co, Ni, Cu, Zn, As, Se, Ag, Sb, Te, Au, Hg, Pb and Bi) hosted within each of the mineral phases. Analysis conditions involved pure helium (He) atmosphere (0,4 L/min), 2.8 J/cm^2^ laser energy, 7 Hz frequency, before transportation to the ICP-MS mixed with argon [Ar (0.95 L/min)] plus nitrogen [N (0,003 L/min)]. Po725 and a synthetic sulphide (^34^S) MASS-1 were used for quality control and as a calibration standard. The matrix-optimized ablation parameters were set at 2.8 J/cm^2^ for laser energy and 7 Hz for the frequency to create a stable signal for suitable sensitivity and to combat laser-induced element fractionation. Due to the fine-grained nature of the material, the ablation spot size used was 20–30 µm at an ablation time of 45 s and a background acquisition of 20 s.

Resultant data were processed using LADR from Norris Scientific^55^. For the correction of the variations in the ablation yielded between the grains and the standards, an internal standard element with a known concentration was utilized^56^. The sulphur (S) content for each sample grain was acquired from SEM analysis beforehand. LA ICP-MS elemental maps on the detrital grain were done with a spot size of 15 µm at a scan speed of 10 µm/sec. The raw data generated was processed and interpolated by Inverse distance weighting (IDW) in ArcMap.

Multivariate analysis or principal component analysis was done using the rounded detrital pyrite trace element data and pyrite trace element data from Archean greenstone belt vestiges within the Kaapvaal craton^24,57,58^. The biplots were generated on R and XLSTAT.

### Mass balance calculations

Mass balance calculations involved determining average gold mineralogical percentages of the heavy mineral fraction displayed in Fig. 2. These percentages values are derived by using the values of the total mass of gold and the total gold hosted in the mineral:

*The total mass of gold (Au *_*total*_*)*: represents the total mass of the gold that is hosted in the heavy mineral fraction for each of the study areas. This value is derived from average gold concentration results determined by aqua regia digestion coupled with ICP-MS (μC _fraction_) and the measured masses from the heavy mineral fraction. For example, the total gold in the heavy mineral fraction from Carletonville tailings samples is calculated as:

1 $${\text{Au}}_{{{\text{total}}}} = \, \left( {\text{mass of fraction}} \right) \, \times \, \left( {\mu {\text{C}}_{{{\text{fraction}}}} } \right),$$

$${\text{Au}}_{{{\text{total}}}} = { 2}{\text{.71}} \times {1}0^{{ - {5}}} {\text{T }} \times { 3}{\text{.36 g}}/{\text{T}},$$

$${\text{Au}}_{{{\text{total}}}} = { 9}{\text{.11 grams of gold}}.$$

*Total gold hosted in the mineral (Au *_*mineral*_*)*: represents the total mass of gold that is hosted within a specific mineral within the heavy mineral fraction. This is derived from the average LA ICP-MS gold concentrations of the specific mineral (μ C_mineral_), the total mass of the heavy mineral fraction and the QEMSCAN-determined bulk percentage of the specific mineral (mineral %). For example, the total gold hosted in pyrite within the heavy mineral fraction from the Carletonville tailings samples is calculated as:

2 $${\text{Au}}_{{{\text{mineral}}}} = \, \left( {{\text{mass of fraction }} \times {\text{ mineral }}\% } \right) \, \times \, \mu {\text{C}}_{{{\text{mineral}}}} ,$$

$${\text{Au}}_{{{\text{pyrite }} = }} \left( {{2}{\text{.71}} \times {1}0^{{ - {5}}} {\text{T }} \times { 63}{\text{.66}}\% } \right) \, \times { 4}.0{\text{9 g}}/{\text{T}},$$

$${\text{Au}}_{{{\text{pyrite}}}} =_{{}} {7}.0{\text{6 grams of gold}}{.}$$

An example of the percentage contribution of gold associated with pyrite to total gold in the heavy mineral fraction from the Carletonville samples is calculated as:

3 $${\text{Percentage association }} = \, \left[ {{\text{Au}}_{{{\text{mineral}}}} } \right]/ \, \left[ {{\text{Au}}_{{{\text{total}}}} } \right] \, \times { 1}00,$$

$${\text{Percentage association pyrite }} = { 7}.0{\text{6 grams}}/{ 9}{\text{.11 grams }} \times { 1}00,$$

$${\text{Percentage association pyrite }} = { 77}{\text{.5}}0 \, \% .$$

An error propagation was used to determine the effects of the mass balance calculation results on the different variable’s uncertainty. The calculation involved the multiplication or division error propagation formula of:

4 $$\frac{{{\upsigma }_{{\text{z}}} }}{{\text{z}}}{ = }\sqrt {\left( {\frac{{{\upsigma }_{{\text{a}}} }}{{\text{a}}}} \right)^{{2}} { + }\left( {\frac{{{\upsigma }_{{\text{b}}} }}{{\text{b}}}} \right)^{{2}} { + }\left( {\frac{{{\upsigma }_{{\text{c}}} }}{{\text{c}}}} \right)^{{2}} { + }\left( {\frac{{{\upsigma }_{{\text{d}}} }}{{\text{d}}}} \right)^{{2}} } ,$$

where a, b, c and d are the variables used in the above mass balance calculations and ơ is the average variables uncertainty value. The summed propagated error percentages (% uncertainty) for each of the study areas was < 3%. Indicating a low propagation of errors that will affect the results of the mass balance calculations.

This mass balance calculation approach (Eqs. (1), (2) & (3)) was also utilized to determine the percentage of heavy metals associations for sulphides displayed in Fig. 4.

## Supplementary Information

Supplementary Figures.

## Data Availability

This study was carried out for the framework of a master of science degree at Stellenbosch University. All the datasets utilized, analyses done and results obtained are accessible from the corresponding author upon reasonable request.
