# Supplementary material for: An underexploited invisible gold resource in the Archean sulphides of the Witwatersrand tailings dumps
Source: Sci Rep. 2023 Feb 22;13:3086. doi: 10.1038/s41598-023-30219-5 (PMC9946953; doi:10.1038/s41598-023-30219-5)
Supplement: Supplementary file 1 — Supplementary Figures. [file 41598_2023_30219_MOESM1_ESM.docx]

**An underexploited invisible gold resource in the Archean sulphides of the Witwatersrand tailings dumps**

**Steve Jason Chingwaru^1*^, Bjorn Von der Heyden^1^ and Margreth Tadie^2^**

1. Department of Earth Sciences, Stellenbosch University, Private Bag X1, Matieland, 7602, South Africa 2. Department of Process Engineering, Stellenbosch University, Private Bag X1, Matieland, Stellenbosch 7602 South Africa

**Supporting online material**


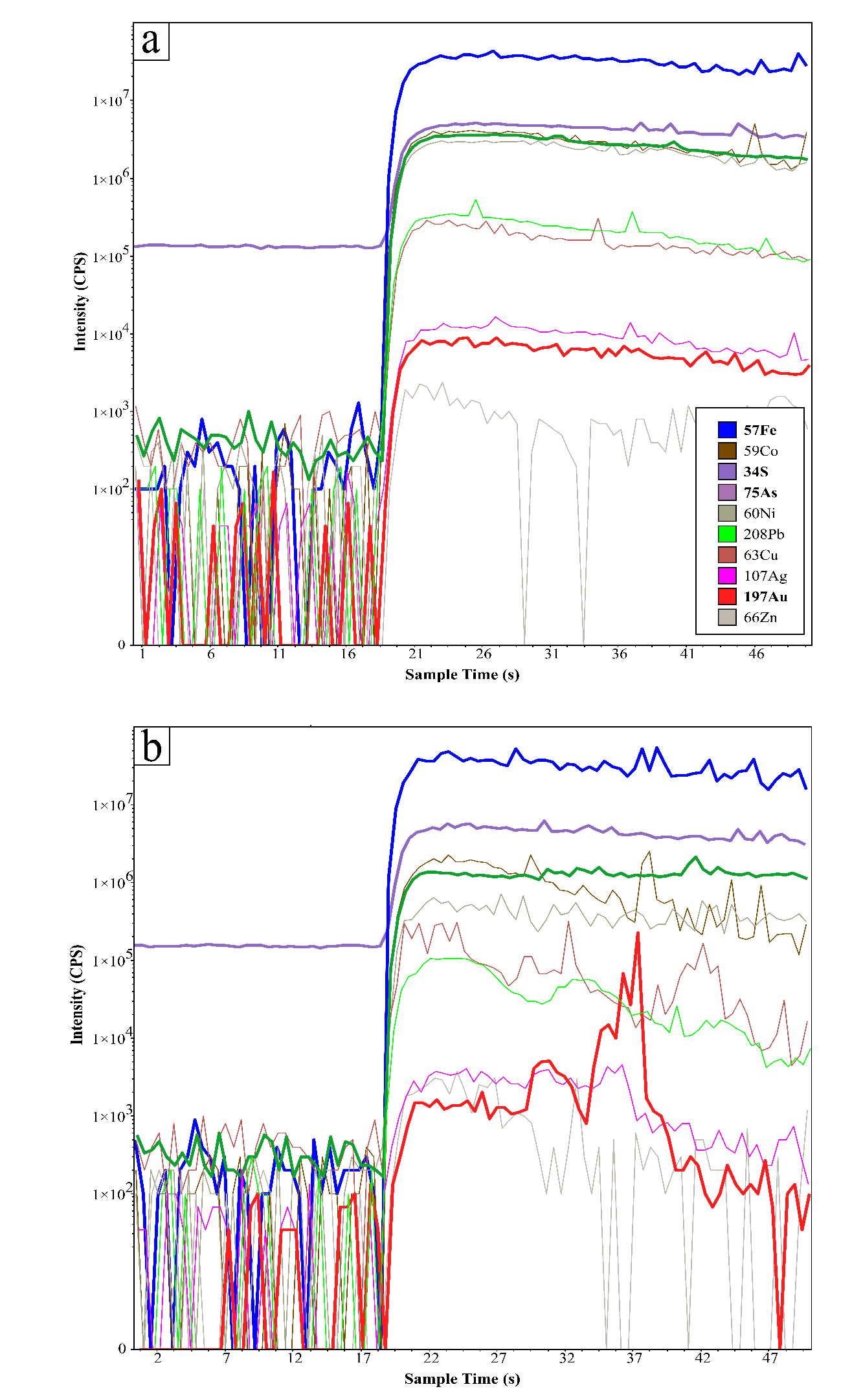


**Figure SI 1**: Time-resolved ablation spots by LA-ICP-MS for two different detrital pyrite grains from the Witwatersrand tailings material. **a)** A pyrite grain with a gold concentration of 10 ppm concentration of gold. Note the intensity of gold is uniform throughout the ablation period which is indicative of gold in solid solution. **b)** A pyrite grain with a gold concentration of 444,76 ppm concentration of gold. Note the intensity of gold is not uniform with a spike at the 37-second mark, this signifies the gold present in this detrital pyrite is likely a nano-particulate inclusion.


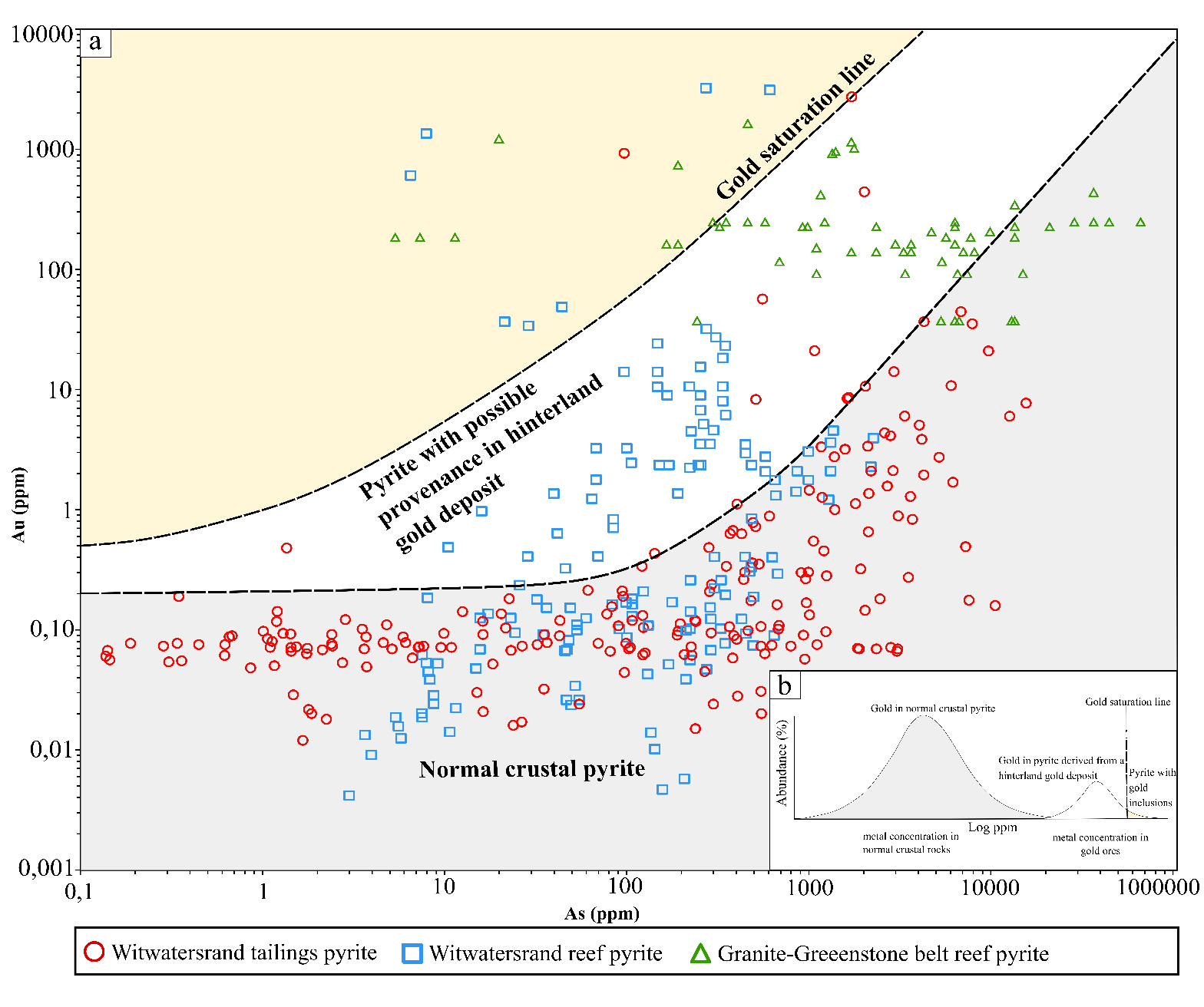


**Figure S1 2: a)** An arsenic/gold scatter plot with concentration points from the Witwatersrand tailings pyrite (this study), Witwatersrand reef pyrite^12,27,38^ and Granite-Greenstone belt auriferous pyrite ^24^. The gold saturation line by Reich *et al*. ^22^ represents the gold/arsenic limit in which gold occurs as free gold particles in pyrite. Inset diagram **(b)** uses the concept of a bi-modal distribution for metal grades, where Clarke values represent the broad main peak (reflecting normal crustal rocks), whereas the second peak represents the metal distribution specifically in ore deposits and occurrences. Here we suggest that our large LA ICP-MS dataset from heterogeneous tailings material provides a representative overview of normal crustal pyrite (e.g., authigenic pyrite, hydrothermal pyrite during modification, as well as detrital pyrite that is not derived from Au deposits), and from pyrite derived from hinterland Au deposits. We suggest that Au/As ratios falling in the grey field represent the signatures of normal crustal pyrite, whereas those in the white and yellow fields may have a provenance deriving from a hinterland Au deposit. The equation separating the normal crustal pyrite from ore-deposit-associated detrital pyrite is given as $\text{C}_{\text{Au}}\text{=0,00}\text{22}\text{. }\text{C}_{\text{As}}\text{+0,2}$. Our LA ICP-MS data targeted the centre of individual pyrite grains to limit any possible signal of increased Au at grain boundaries emanating from post-depositional hydrothermal modification (i.e., following the modified paleo-placer model). The high number of “Witwatersrand reef pyrite” samples falling within the white field of the plot predominantly correspond to data points from Large *et al.,*^12^ and Large & Maslennikov ^38^ of hydrothermal pyrite overgrowths on the rims of detrital pyrite and were used to infer both placer and hydrothermal process regarding the enrichment of gold content Carbon Leader Reef.

**
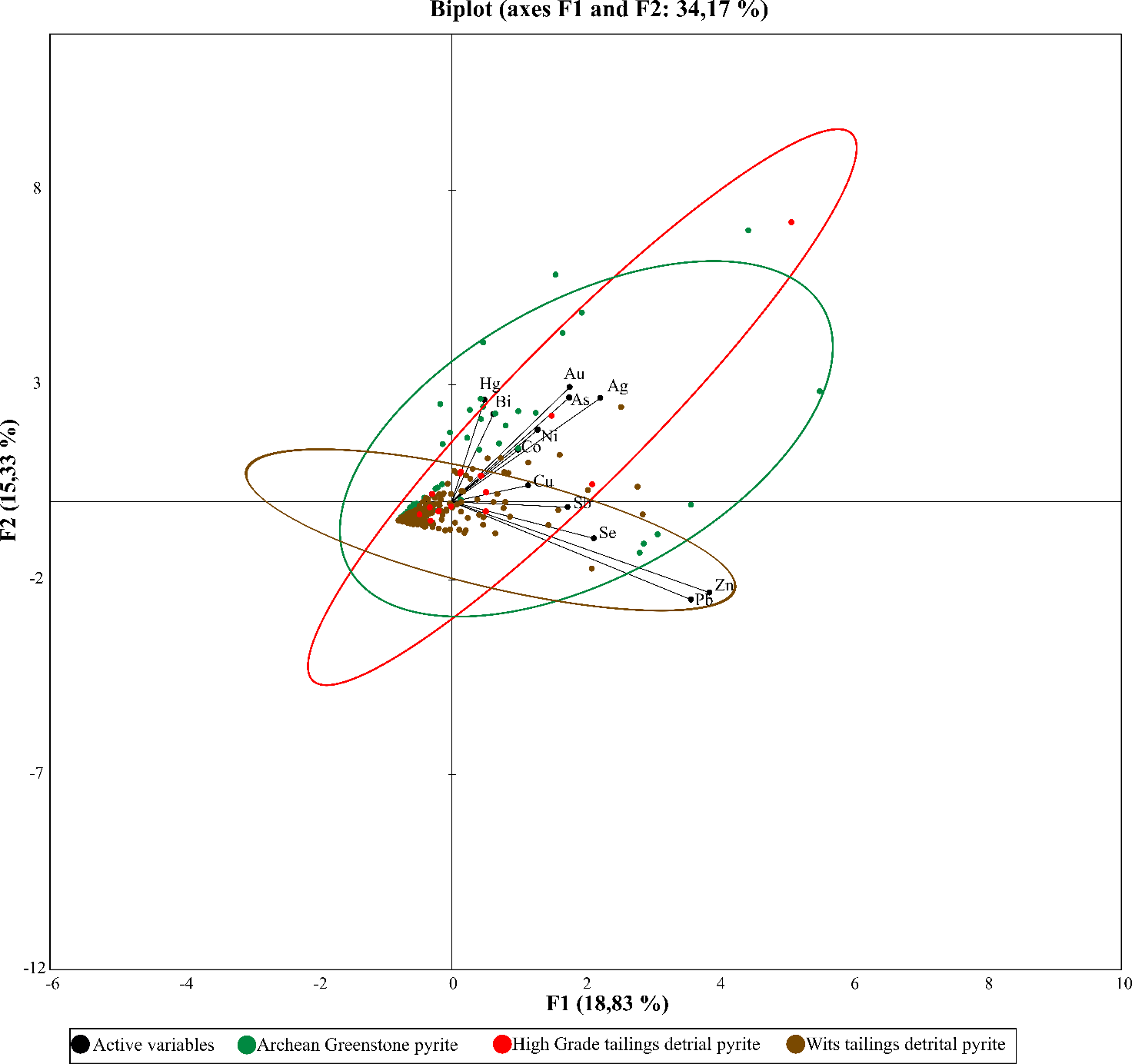
**

**Figure S1 3:** A multivariable analysis or principal component analysis (PCA) is used to determine the relationship between the detrital pyrite trace element data generated in this study with pyrite trace element data from Archean greenstone belt vestiges within the Kaapvaal craton ^24,58,59^. The axes F1 and F2 were chosen to be displayed on the biplot, as these axes' dimensions represent the majority of the initial multivariable trace element data.

The active variables (black vectors) indicated the correlation between the different trace elements datasets used for the multivariate analysis. The lengths of the variables in the biplot represent the representativeness and quality of trace element data within the investigated axes dimensions (F1 and F2). This means Au, As, Ag, Zn and Pb trace element data is best represented in the F1 and F2 dimensions. Gold and arsenic show a high positive correlation due to the vectors forming narrow angles. This indicates that arsenic is a relevant indicator of invisible gold within this trace element data set.

The high-grade gold tailings detrital pyrite that overlaps with the Archean greenstone pyrite in figure 3a are plotted in different symbology from the moderate to low-grade gold tailings detrital pyrite. The two high-grade detrital tailings pyrite (red dots in figure S1.) plot within the Archean Greenstone pyrite field, with one outlier plotting closely with one Archean greenstone pyrite outlier. Two high-grade Au tailings pyrite are plotting very close to the moderate Au-grade pyrite boundary. The biplot indicates there is some relation between the high-grade pyrite in the tailings and the pyrite from the Archean Greenstone Belt vestiges.
